# Establishment of Hairy Root Cultures by *Agrobacterium Rhizogenes* Mediated Transformation of *Isatis Tinctoria* L. for the Efficient Production of Flavonoids and Evaluation of Antioxidant Activities

**DOI:** 10.1371/journal.pone.0119022

**Published:** 2015-03-18

**Authors:** Qing-Yan Gai, Jiao Jiao, Meng Luo, Zuo-Fu Wei, Yuan-Gang Zu, Wei Ma, Yu-Jie Fu

**Affiliations:** 1 Key Laboratory of Forest Plant Ecology, Ministry of Education, Northeast Forestry University, Harbin, 150040, PR China; 2 Collaborative Innovation Center for Development and Utilization of Forest Resources, Harbin, 150040, PR China; 3 State Key Laboratory of Tree Genetics and Breeding, Northeast Forestry University, Harbin, 150040, PR China; 4 School of Pharmaceutical, Heilongjiang University of Chinese Medicine, Harbin, 150040, PR China; University of Wisconsin-Milwaukee, UNITED STATES

## Abstract

In this work, *Isatis tinctoria* hairy root cultures (ITHRCs) were established as an alternative source for flavonoids (FL) production. *I*. *tinctoria* hairy root line V was found to be the most efficient line and was further confirmed by the PCR amplification of *rol*B, *rol*C and *aux*1 genes. Culture parameters of ITHRCs were optimized by Box-Behnken design (BBD), and eight bioactive FL constituents (rutin, neohesperidin, buddleoside, liquiritigenin, quercetin, isorhamnetin, kaempferol and isoliquiritigenin) were quali-quantitatively determined by LC-MS/MS. Under optimal conditions, the total FL accumulation of ITHRCs (24 day-old) achieved was 438.10 μg/g dry weight (DW), which exhibited significant superiority as against that of 2 year-old field grown roots (341.73 μg/g DW). Additionally, *in vitro* antioxidant assays demonstrated that ITHRCs extracts exhibited better antioxidant activities with lower IC_50_ values (0.41 and 0.39, mg/mL) as compared to those of field grown roots (0.56 and 0.48, mg/mL). To the best of our knowledge, this is the first report describing FL production and antioxidant activities from ITHRCs.

## Introduction

*Isatis tinctoria* L. (woad), the biennial herb of Brassicaceae family, is a popular medicinal crop widely cultured in Europe and Asian countries [1]. Its roots (Radix isatidis) known as Ban-Lan-Gen has been used in Traditional Chinese Medicine (TCM) for hundreds of years for the clinical treatment of pestilence, epidemic hepatitis and infections [2], especially for influenza such as severe acute respiratory syndrome (SARS) and H1N1 [3, 4]. Alkaloids, phenylpropanoids and terpenoids are recognized as the principle active ingredients of Radix isatidis [5]. Among them, alkaloids always attract much attention, and are validated to be responsible for various bioactivities [6]. Nevertheless, phenylpropanoids mainly comprised of flavonoids (FL) have been identified as anti-inflammatory and antiviral constituents, and also are involved in the major drug actions of Radix isatidis [3, 7]. In this context, the interest and market demand of FL from Radix isatidis is increasing more and more.

Due to the unreliability on harvest of phytochemicals from natural resources and the complexity in producing natural products through chemical synthesis, one has to look for an environment friendly and renewable production system to fulfill the need of food and pharmaceutical industries. Plant cell culture technology emerging as an attractive alternative system, can continuously provide high-value ingredients independent of geographical, climatic or environmental variations and constraints [8, 9]. Over the past years, plant cell suspension cultures for the production of secondary metabolites have been hampered by several limitations, such as low yields of desired compounds, expensive culturing process, application of phytohormones, heterogeneous cell types, lack of storage tissue, and products easily degraded by the enzymes released in the media [9, 10]. One way around this problem has been the development of specialized differentiated or plant organ cultures instead of cell suspension cultures, best exemplified by *Agrobacterium rhizogenes*-based hairy root cultures (HRCs). HRCs induced by the infection of wounded plant tissues with *A*. *rhizogenes* bearing the root-inducing (Ri) plasmid, possess comparable biosynthetic capacity of secondary metabolites to native plant roots with advantages of fast growth rates independent of phytohormones, genetic and biochemical stability, long-term preservation, and sizable biomass production [11]. More importantly, HRCs often accumulate phytochemicals at a higher level as against undifferentiated cell suspension cultures [12]. Herein, it is believed that the transformation of *I*. *tinctoria* by *A*. *rhizogenes* could result in hairy root lines with the potential to biosynthesize FL for research or food, agricultural and pharmaceutical applications.

The present study demonstrated a protocol for the development of *A*. *rhizogenes*-mediated hairy root system in *I*. *tinctoria* to produce valuable FL. The high-productive *I*. *tinctoria* hairy root line (ITHRL) was initially screened followed by the molecular characterization. Afterwards, the culture conditions of *I*. *tinctoria* HRCs (ITHRCs) were optimized systematically for the efficient production of FL, which may provide valuable data for industrial scale-up applications in bioreactors. Subsequently, eight FL constituents from ITHRCs including rutin (RUT), neohesperidin (NEO), buddleoside (BUD), liquiritigenin (LIQ), quercetin (QUE), isorhamnetin (ISR), kaempferol (KAE) and isoliquiritigenin (ISL) were quali-quantitatively determined by LC-MS/MS. Moreover, considering that antioxidant activities of FL are of great interest in food, cosmetic and pharmaceutical fields, antioxidant capacities of ITHRCs extracts were also evaluated. Furthermore, the predominance of ITHRCs was eventually summarized as against *I*. *tinctoria* field grown roots (ITFGRs). In light of the presented results, ITHRCs may offer a promising and continuous product platform for naturally-derived, high quality and valuable nutraceuticals.

## Materials and Methods

### Seed sterilization and germination

Mature seeds of *I*. *tinctoria* were generously provided by Rongquan Medicine Plant Co. Ltd., Daqing, Heilongjiang Province, China. FL standards including RUT, NEO, BUD, LIQ, QUE, ISR, KAE and ISL were purchased from Weikeqi Biological Technology Co. Ltd. (Sichuan province, China). Other reagents of either analytical or optical grades were obtained from Beijing Chemical Reagents Co. (Beijing, China). To produce *I*. *tinctoria* aseptic plantlets, seeds were surface sterilized with 70% (v/v) ethanol for 45 s and 4% (v/v) sodium hypochlorite solution for 8 min, and then rinsed 5 times with sterilized water. After that, seeds were immediately germinated on Murashige and Skoog (MS)-based solid medium and incubated in a growth chamber under 16/8 h of light/dark photoperiod at 25 ± 1°C. The petioles of 3-week-old seedling were used as explants for hairy root induction.

### Induction of hairy roots

The explants (excised petioles) were pre-incubated on half-strength MS (MS/2)-based solid medium for 2 days prior to infection. *A*. *rhizogenes* strain LBA9402 was grown overnight in the dark at 28 ± 1°C with shaking (180 rpm) in liquid Luria-Bertani (LB) medium. The bacterial cells were collected by centrifugation at 2000 rpm for 10 min and resuspended in MS/2-based liquid medium containing vitamins and sucrose (3.0%) for inoculation. Thereafter, the explants were immersed into the overnight grown bacterial suspension of *A*. *rhizogenes* strain LBA9402 (OD_600 nm_ = 0.6–0.8) for 6 min, dry-blotted on sterile filter paper, and incubated in the dark at 25 ± 1°C on MS/2-based solid medium supplemented with 1 mM arginine and 125 μM acetosyringone. After 2 days of co-cultivation, the explants were transferred to hormone-free MS/2-based solid medium containing sodium cefotaxim (300 mg/L) to eliminate the residual bacteria, and incubated in the dark at 25 ± 1°C. Putative transgenic hairy roots were observed emerging from the wound sites of explants within 16 days of incubation. Subsequently, the root initials were isolated from the explants and sub-cultured on fresh hormone free MS/2-based solid medium at 25 ± 1°C in the dark every 2 weeks. During the sub-cultivation, the concentration of antibiotic was gradually decreased and finally omitted until the bacteria were eliminated completely.

### Molecular characterization of hairy roots

Integration of T-DNA responsible for hairy roots formation was confirmed by PCR analysis using *rol*B, *rol*C, *aux*1 and *vir*D specific primers according to previous reports [13, 14]. Genomic DNA was isolated from the selected transformed hairy root line using a DNeasy Plant Mini Kit (Tiangen China) following the manufacturer’s instructions. PCR amplification of *rol*B, *rol*C and *aux*1 genes was performed by a S1000 thermal cycler (Bio-Rad, Hercules, CA) according to the program supplemented in S1 Table. Genomic DNA isolated from *I*. *tinctoria* aseptic plantlets and Ri-plasmid (pRi) DNA from *A*. *rhizogenes* strain LBA9402 were used as negative and positive controls, respectively. PCR products were analyzed by electrophoresis on a 2.5% (w/v) agarose-ethidium bromide gel along with 1000 bp DNA marker.

### Optimization of culture conditions

After the high-productive ITHRL identified, a certain amount of hairy roots was transferred into 250 mL Erlenmeyer flasks containing 150 mL of MS/2 liquid medium and incubated on a rotary shaker (120 rpm) at an appropriate temperature in the dark. ITHRCs were harvested by filtration after a certain period of cultivation and dried in a vacuum drier at 60°C till constant weight. Thereafter, the biomass DW and total flavonoids (TFL) content (the sum amount of RUT, NEO, BUD, LIQ, QUE, ISR, KAE and ISL) were measured and determined, respectively.

In order to obtain the optimal biomass production and FL accumulation during the culture process, BBD was applied to survey effects of four independent key variables (culture temperature 20–30°C, sucrose concentration 2–4%, inoculum size 0.4–1% and harvest time 18–30 days) on dependent variables (biomass DW and TFL content). The inoculum size was calculated based on the fresh weight of hairy roots. The regression analysis was carried out to evaluate the response function as a quadratic polynomial:

$$Y=\beta_{0}+\overset{k}{\underset{j=1}{\sum^{\text{​}}}}\beta_{j}X_{j}+\overset{k}{\underset{j=1}{\sum^{\text{​}}}}\beta_{jj}X_{j}^{2}+\sum^{\text{​}}\sum_{i<j}^{}\beta_{ij}X_{i}X_{j}\left(k=4\right)$$

Where, *Y* is the predicted response; *β*_*0*_, *β*_*j*_, *β*_*jj*_ and *β*_*ij*_ are regression coefficients for intercept, linearity, square and interaction, respectively; *X*_*i*_ and *X*_*j*_ are independent coded variables; and *k* represents the number of variables. The actual and coded levels of independent variables used in the experimental design are summarized in S2 Table. The experiment data were analyzed statistically with Design-Expert 7.0 (State-Ease, Inc., Minneapolis MN). Analyses of variance (ANOVA) were performed to calculate and simulate the optimal values of the tested parameters.

### Extraction of FL

The powders (0.5 g DW) of hairy roots and 2 year-old field grown roots were extracted with 80% ethanol solution (20 mL) in an ultrasonic bath for 120 min. For the complete extraction of FL, the above procedure was repeated for 3 cycles. Subsequently, the supernatant extracts were combined and condensed to dryness using a rotary evaporator under vacuum with oil pump at 45°C. Thereafter, the resulting extracts were re-dissolved in 20 mL of acetonitrile (HPLC grade) and then filtered through a 0.45 μm membrane for LC-MS/MS analysis.

### LC-MS/MS analysis of FL

An Agilent 1100 series HPLC (Agilent, San Jose, CA, USA) coupled to an API 3000 triple tandem quadrupole MS (Applied Biosystems, Concord, Ontario, Canada) equipped with a Phenomenex Gemini C18 110A reversed-phase column (250 mm × 4.6 mm I.D., 5 μm) was applied for the analysis of FL. The binary mobile phase consisted of acetonitrile (A) and 0.001% formic acid aqueous solution (B) using the gradient program as follows: 0–5 min, 40–50% (A); 5–13 min, 50–60% (A); 13–15 min, 60–68% (A); 15–16 min, 68–40% (A); and 16–18 min, 40% (A). The column temperature was maintained at 30°C, the flow rate 1.0 mL/min and the injection volume 10 μL. Mass spectra of analyses were performed in the selected reaction monitoring (SRM) transitions with an electrospray ionization source operating in the negative ion mode. Analytical conditions were optimized and summarized in S3 Table. The content of each target compound was calculated by the corresponding calibration curve and reported as the microgram per gram of roots DW.

### Determination of antioxidant activities

Antioxidant activities of extracts from ITHRCs and ITFGRs were assessed using the DPPH radical-scavenging assay and β-carotene/linoleic acid bleaching test. Scavenging activities of samples towards DPPH radicals were determined according to the pervious method reported by Wu et al. [15]. Lipid antioxidant activities of samples towards β-carotene/linoleic acid were evaluated in accordance with the method described by Wu et al. [16]. VC and BHT were used as the synthetic antioxidants. Antioxidant activities of samples were reported as IC_50_ values, which were calculated using the logarithmic regression curves for DPPH radical scavenging ratio or β-carotene bleaching inhibition percentages (%) versus the concentrations of samples (mg/mL).

### Statistical analysis

Results were expressed as means ± standard deviations. The data were statistically analyzed using the SPSS statistical software, version 17.0 (SPSS Inc, Chicago, Illinois, USA). Differences between means were determined by analysis of variance (ANOVA) with Duncan’s test on the level of significance declared at *P* < 0.05.

## Results and Discussion

### Establishment of *I*. *tinctoria* hairy roots

**Fig 1:**
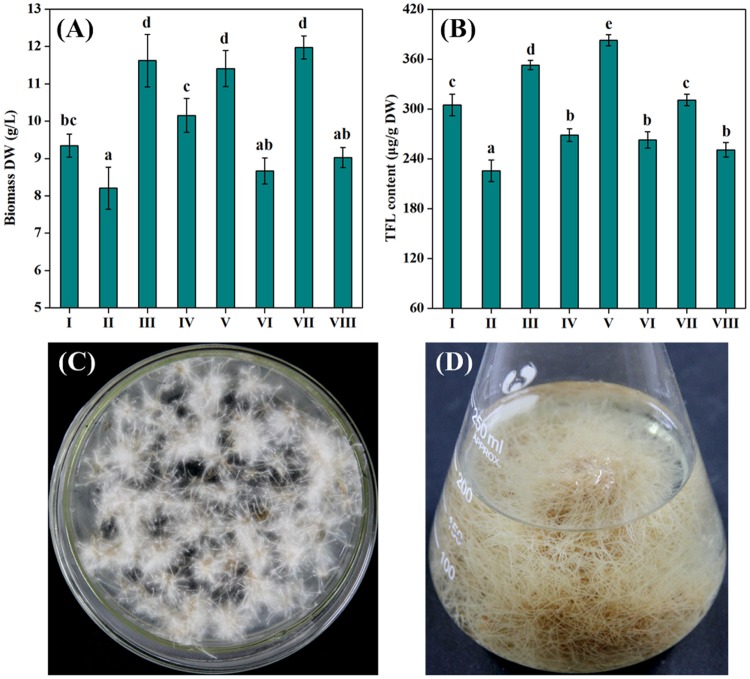
Selection of high-productive ITHRL. **(A)** Biomass production and **(B)** FL accumulation of eight selected ITHRL (I-VIII) after 3 weeks of cultivation in MS/2 liquid medium (initial pH 5.8, inoculum size 0.5%, culture temperature 25°C and sucrose concentration 3%). **(C)** Cultivation of ITHRL V on MS/2 solid medium and **(D)** its prolific growth in MS/2 liquid medium. Mean ± SD values not sharing the same lowercase letters are significantly different (*P* < 0.05).

Based on results of preliminary experiments (data not shown), the petiole sections of *I*. *tinctoria* were provided as the potential donor explants for the genetic transformation with *A*. *rhizogenes* strain LBA9402. The successful transformation was indicated by the direct emergence of roots, formation of callus or gall-like structures from the wounded sites of explants. The optimal transformation rate (76.67%) was obtained when 3 week-old petiole explants were co-cultured for 2 days with the supplementation of 125 μM acetosyringone and 1.5 mM arginine. The transformed roots would emerge at the wounded sites of explants after 3 weeks of cultivation. Subsequently, the independently transformed hairy root lines were excised and sub-cultured on MS/2 solid medium with antibiotics to eliminate bacteria. As presented in Fig. 1C, the established *I*. *tinctoria* hairy roots exhibited the typical morphological characteristics with vigorous growth on phytohormone-free medium, lack of geotropism and extensive lateral branching.

### Selection of high-productive hairy root line

The fundamental basis of an efficient HRCs process is the development of an appropriate hairy root line that maximizes both growth rate and product yield [9, 17]. Generally, different derived hairy root lines often exhibit considerable variation in biomass and metabolite production owing to the site uncertainty of T-DNA integration into the host plant genome [18, 19]. Therefore, selection of the high-productive hairy root line is extremely important in this work. Accordingly, eight candidate lines (ITHRL I–VIII) with vigorously growing phenotype were evaluated by comparing their performance of biomass production and FL accumulation. As shown in Fig. 1A, B, ITHRL III, V and VII were categorized as high-productive lines in terms of biomass DW (11.41–11.97 g/L) and FL content (310.83–382.71 μg/g DW) as compared to the others. However, ITHRL V exhibited the most intensive ability of FL biosynthesis among them. Consequently, ITHRL V was demonstrated to be the optimal hairy root line used for the subsequent work. Moreover, the cultivation of ITHRL V on MS/2 solid medium and its prolific growth in MS/2 liquid medium are shown in Fig. 1C, D, respectively.

### PCR identification of hairy roots

**Fig 2:**
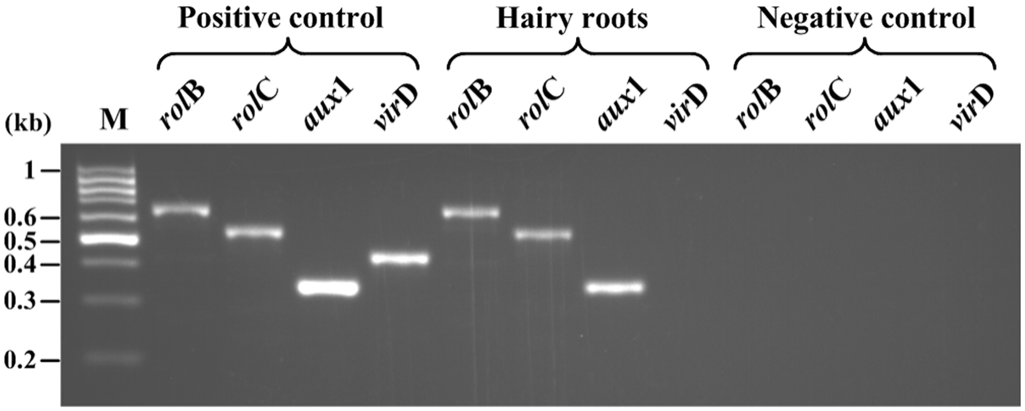
Molecular characterization of ITHRL V. PCR analysis of *rol*B (670 bp), *rol*C (534 bp), *aux*1 (350 bp) and *vir*D (438 bp) genes in hairy roots (genomic DNA extracted from the transformed ITHRL V), positive control (Ri-plasmid DNA extracted from *A*. *rhizogenes* LBA 9402) and negative control (genomic DNA extracted from *I*. *tinctoria* aseptic plantlets). PCR products were analyzed by electrophoresis on a 2.5% (w/v) agarose-ethidium bromide gel along with 1000 bp DNA marker.

Based on the typical morphologies, it could be concluded that the selected ITHRL V was the hairy root, but that should have to be confirmed at the molecular level. Factually, the integration of Ri T-DNA into the genome of plant cells caused the formation of hairy roots, in which *rol* and *aux* genes were harbored [20]. Herein, PCR-based analysis of *rol*B, *rol*C, *aux*1 and *vir*D genes was conducted to assess the genetic transformation status of ITHRL V. In detail, the *rol*B and *rol*C genes (located at the Ri TL-DNA segment) and the *aux*1 gene (located at the Ri TR-DNA segment), were diagnostic for T-DNA integration into the host genome of ITHRL V. Additionally, the *vir*D gene (located outside the Ri T-DNA segment) was used to verify the complete absence of *A*. *rhizogenes* in ITHRL V. As exhibited in Fig. 2, PCR analysis of the reference genes showed that ITHRL V carried *rol*B, *rol*C and *aux*1 genes in its genome but not the *vir*D gene, which confirmed that the genetic transformation in ITHRL V and the complete elimination of bacteria.

### Optimization of culture conditions

**Fig 3:**
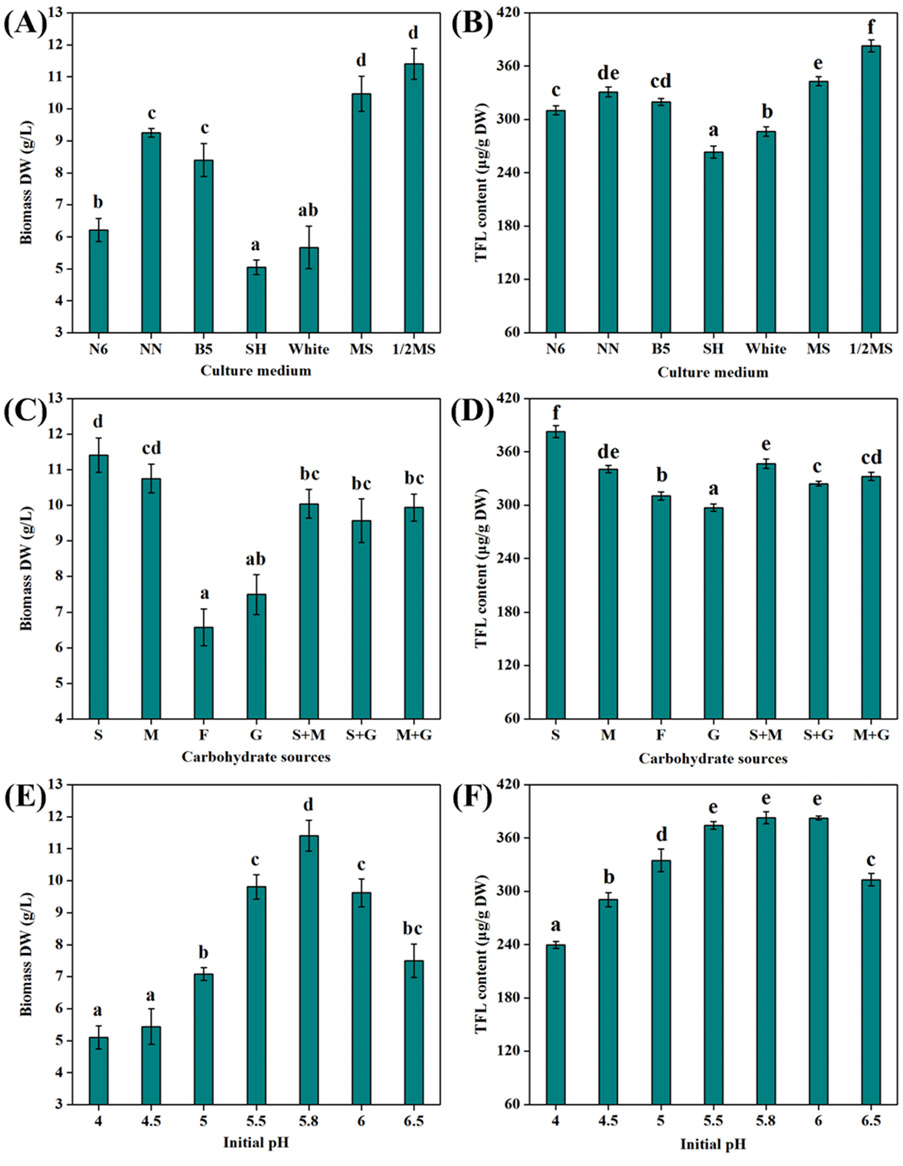
Optimization of culture media, carbohydrate sources and initial pH. Effect of different culture media on **(A)** biomass production and **(B)** FL accumulation of ITHRCs after 3 weeks of cultivation (initial pH 5.8, culture temperature 25°C, sucrose concentration 3% and inoculum size 0.5%). Effect of different carbohydrate sources on **(C)** biomass production and **(D)** FL accumulation of ITHRCs after 3 weeks of cultivation (MS/2 medium, initial pH 5.8, culture temperature 25°C, inoculum size 0.5% and sugar concentration 3%). Effect of initial pH on **(E)** biomass production and **(F)** FL accumulation of ITHRCs after 3 weeks of cultivation (MS/2 medium, culture temperature 25°C, inoculum size 0.5% and sucrose concentration 3%). S: Sucrose; M: Maltose; F: Fructose; G: Glucose. Mean ± SD values not sharing the same lowercase letters are significantly different (*P* < 0.05).

Once the high-productive hairy root line was established, the optimization of culture conditions can further improve the product yield. Generally, a suitable type of culture medium was beneficial for cell/organ growth and metabolite biosynthesis [19]. As presented in Fig. 3A, B, MS/2 liquid medium was favorable for biomass production and FL accumulation as against other media. Moreover, sugars acting as both energy sources and signaling molecules always affect the growth and metabolism of plant cell/organ cultures [21]. As shown in Fig. 3C, D, sucrose was found to be the suitable carbon source for root growth and FL biosynthesis. Furthermore, it is reported that the initial pH of culture medium can significantly affect nutrient uptake as well as enzymatic and hormonal activities in plant cell/organ cultures [22]. As exhibited in Fig. 3E, F, the initial pH at 5.8 gave the maximum values of biomass DW and TFL content. Additionally, the number of experiments necessary for optimizing culture conditions can be reduced by following statistical experimental design due to the tedious process. Herein, the remaining key parameters including culture temperature, sucrose concentration, inoculum size and harvest time were optimized by BBD. The experimental design matrix and the relevant data are illustrated in S2 Table.

### Fitting the mathematical model

ANOVA results of the quadratic models are presented in S4 Table. The highly significant levels of the models (*P* < 0.0001), not significant “lack of fit” (*P* > 0.05) and desirable determination coefficients (*R* ^2^ ≥ 0.9754) suggested that both the built mathematical models were precise and applicable. The second-order polynomial models were applied to express the biomass production and FL accumulation of ITHRCs as the following equations:

$$Y_{Bio}=12.35-0.28X_{1}+0.03X_{2}+0.49X_{3}-0.012X_{4}+0.01X_{1}X_{2}-0.02X_{1}X_{3}-0.02X_{1}X_{4}+0.09X_{2}X_{3}-0.02X_{2}X_{4}-0.35X_{3}X_{4}-2.6X_{1}^{2}-0.75X_{2}^{2}-1.15X_{3}^{2}-0.83X_{4}^{2}$$

$$Y_{TFL}=428.8-9.73X_{1}+5.05X_{2}+8.95X_{3}-2.63X_{4}-0.9X_{1}X_{2}-2.87X_{1}X_{3}-2.3X_{1}X_{4}-1.98X_{2}X_{3}+9.38X_{2}X_{4}-0.6X_{3}X_{4}-82.94X_{1}^{2}-23.43X_{2}^{2}-45.35X_{3}^{2}-21.87X_{4}^{2}$$

Where *Y*_*Bio*_ and *Y*_*TFL*_ were the biomass DW (g/L) and TFL content (μg/g DW), respectively; *X*_1_ was the culture temperature (°C); *X*_2_ was the sucrose concentration (%), *X*_3_ was the inoculum size (%) and *X*_4_ was the harvest time (days).

### Analysis of the response contour

**Fig 4:**
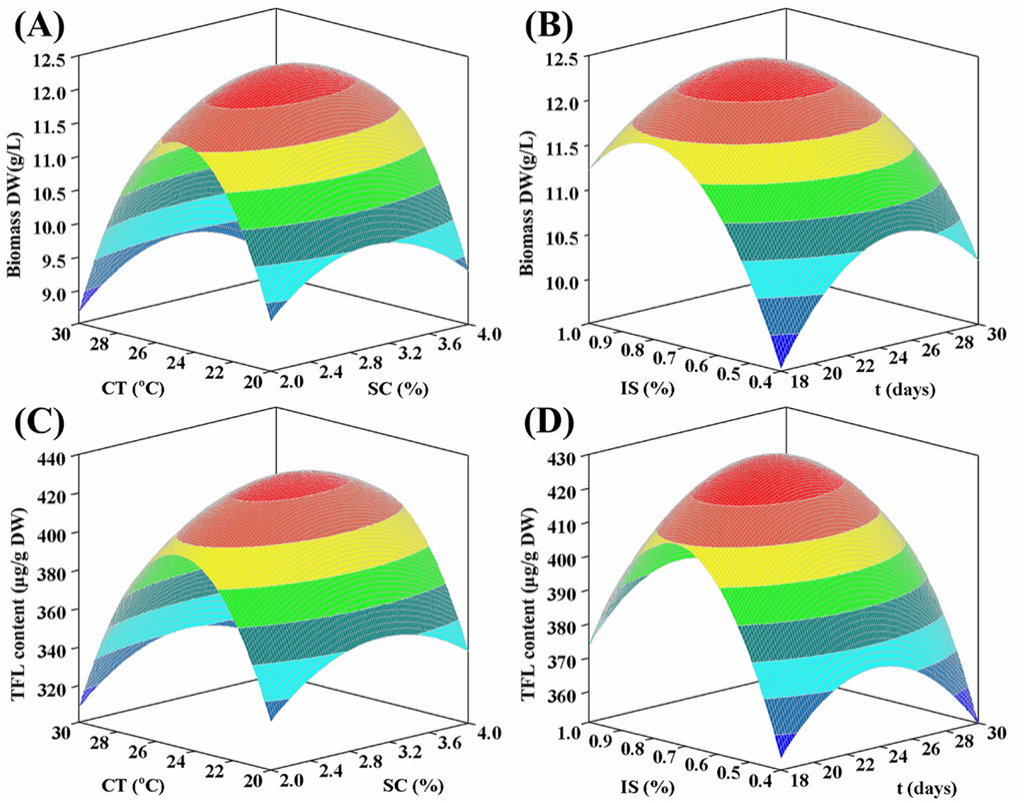
Response contours for biomass production and FL accumulation of ITHRCs in MS/2 liquid medium. **(A)** Varying culture temperature (X axis,°C) and sucrose concentration (Y axis, %) on biomass DW (Z axis, g/L). **(B)** Varying inoculum size (X axis, %) and harvest time (Y axis, days) on biomass DW (Z axis, g/L). **(C)** Varying culture temperature (X axis,°C) and sucrose concentration (Y axis, %) on TFL content (Z axis, μg/g DW). **(D)** Varying inoculum size (X axis, %) and harvest time (Y axis, days) on TFL content (Z axis, μg/g DW).

As shown in Fig. 4A, C, both culture temperature and sucrose concentration exhibited double impacts on biomass DW and TFL content. Generally, the growth and metabolism of hairy roots were enhanced with increasing temperature, while high temperature (25–30°C) would cause an irreversible damage of some related proteins or enzymes in plant cells/organs [23], thus resulting in the decrease in biomass DW and TFL content. Additionally, sucrose concentration around 3.0% favored the biomass production and TFL accumulation at a given temperature. It is known that sucrose has dual roles as carbon source and osmotic agent in plant cells/organs cultures [19]. Generally, more consumption of sucrose would benefit root growth and metabolite biosynthesis. However, the osmolality of culture medium under high sucrose concentration (3.0–4.0%) could cause the loss of cell viability by the dehydration and promote the diffusion of phytochemicals from root tissues into the liquid medium, thus leading to the low biomass DW and TFL content. As presented in Fig. 4B, D, biomass DW and TFL content increased obviously with the extension of inoculum size from 0.4% to 0.8% at a fixed harvest time, but decreased significantly afterward being ascribed to the limitation of nutrients, oxygen levels and volume of culture flasks [24]. Furthermore, at a given inoculum size, biomass DW and TFL content increased with raising time initially but decreased gradually beyond 25 days owing to the depletion of medium nutrients and the liquid mass-transfer limitations [25].

### Verification of the predictive models

By the aid of the above mathematical models, the optimal conditions for biomass DW and TFL content in ITHRCs were calculated as follows: culture temperature 24.71°C, sucrose concentration 3.06%, inoculum size 0.75% and harvest time 23.74 days. Considering the actual operation, temperature and time were modified to 24.7°C and 24 days, respectively. To validate the reliability of these predictive parameters, six sequential experiments adopting ITHRL V were performed under the above optimized conditions. The optimal values of biomass DW (12.53 ± 0.26 g/L) and TFL content (438.10 ± 3.46 μg/g DW) were very close to those (12.41 g/L and 429.66 μg/g DW) forecasted by the theoretical models. Consequently, the culture conditions achieved by BBD were reliable and practical.

### LC-MS/MS analysis of FL

**Fig 5:**
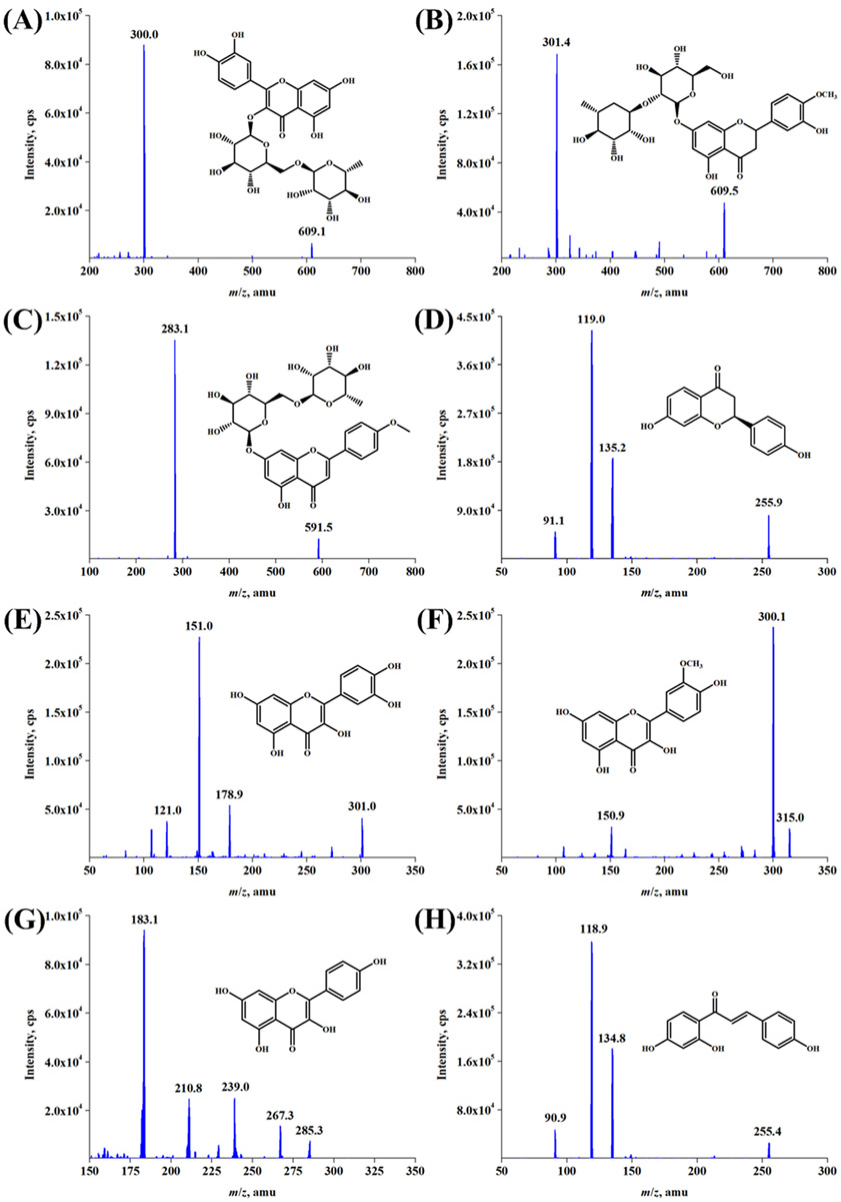
ESI-MS/MS analysis of FL constituents originated from ITHRCs. **(A)** RUT, **(B)** NEO, **(C)** BUD, **(D)** LIQ, **(E)** QUE, **(F)** ISR, **(G)** KAE and **(H)** ISL. In all cases, X axis was indicated as mass-to-charge ratio (*m/z*, amu) and Y axis was indicated as ion abundance (intensity, cps).

**Fig 6:**
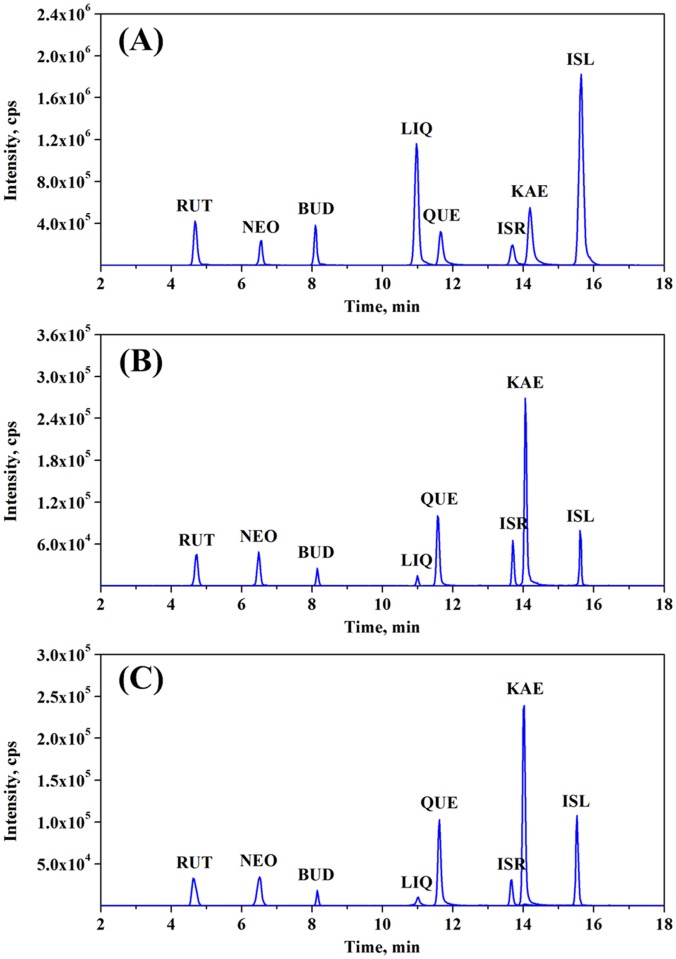
Representative LC-MS/MS with SRM total ion chromatograms. **(A)** Standards, extracts form **(B)** 24 day-old ITHRCs and **(C)** 2 year-old ITFGRs. In all cases, X axis was indicated as retention time (time, min) and Y axis was indicated as ion abundance (intensity, cps).

In this work, eight FL constituents (RUT, NEO, BUD, LIQ, QUE, ISR, KAE and ISL) originating from ITHRCs were identified by ESI-MS/MS analysis (Fig. 5). RUT (retention time 4.69 min) produced a precursor ion of *m/z* 609.1 [M-H]^-^, which fragmented into the main product ion of *m/z* 300.0 [M-H-rutinose]^-^ (Fig. 5A). Likewise, the mass spectra fragmentation patterns of NEO, BUD, LIQ, QUE, ISR, KAE and ISL are exhibited in Fig. 5B-H, respectively. All information of mass spectra of these compounds was consistent with the reported data [26, 27] as well as that of standard compounds, which confirmed that the established ITHRCs could indeed produce the eight FL constituents. Additionally, the precursor/product ion combinations of these target analytes with the highest intensity were chosen for the quantification via LC-MS/MS analysis with SRM mode. The relevant operating parameters are optimized and shown in S3 Table, and the representative chromatograms are presented in Fig. 6.

The corresponding contents of RUT, NEO, BUD, LIQ, QUE, ISR, KAE and ISL in ITHRCs were calculated as 94.13 ± 1.65, 50.97 ± 0.73, 13.78 ± 0.42, 4.71 ± 0.39, 53.28 ± 1.56, 83.79 ± 0.94, 134.50 ± 2.38, 2.94 ± 0.25, μg/g DW, which were comparable to or greater than those from 2 year-old ITFGRs (69.88 ± 2.79, 41.24 ± 1.61, 7.19 ± 0.65, 1.37 ± 0.18, 60.84 ± 2.37, 56.09 ± 1.57, 100.35 ± 3.42, 3.77 ± 0.36, μg/g DW, correspondingly). Overall, ITHRCs accumulated up to 1.28 times more TFL (438.10 ± 3.46 μg/g DW) than that of ITFGRs (341.73 ± 4.85 μg/g DW), confirming that the T-DNA of *A*. *rhizogenes* played an important role in boosting the production of FL in ITHRCs. Evidently, T-DNA associated with *rol* genes in the host genome of HRCs are thought to induce and enhance the biosynthesis of plant secondary metabolites by turning on the transcription of defense genes [14, 28, 29]. It can be inferred that *rol*B and *rol*C genes existed in ITHRCs (Fig. 2) are likely to be stimulators for the activation of secondary metabolism to improve FL biosynthesis.

### Antioxidant activities

**Fig 7:**
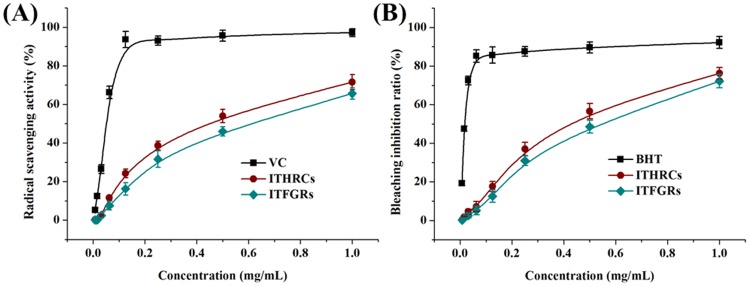
Antioxidant activities assessed by DPPH radical scavenging assay and β-carotene/linoleic acid bleaching test. **(A)** Antioxidant activities of extracts from ITHRCs and ITFGRs assessed by DPPH radical scavenging assay. X axis was indicated as the concentration of extracts (mg/mL) and Y axis was indicated as the DPPH radical scavenging activity (%). **(B)** Antioxidant activities of extracts from ITHRCs and ITFGRs assessed by β-carotene/linoleic acid bleaching test. X axis was indicated as the concentration of extracts (mg/mL) and Y axis was indicated as the β-carotene bleaching inhibition ratio (%).

As shown in Fig. 7A, B, the antioxidant activities of extracts from ITHRCs and ITFGRs appeared obviously as the dose-dependent relationships. However, ITHRCs extracts exhibited superior efficacies in terms of anti-radical (IC_50_ value of 0.41 mg/mL) and lipid peroxidation inhibitory (IC_50_ value of 0.39 mg/mL) as against those of AMFGRs (IC_50_ values of 0.56 and 0.48, mg/mL, respectively). Additionally, the IC_50_ values of VC (the reference in DPPH radical-scavenging assay) and BHT (the reference in β-carotene/linoleic acid bleaching test) were 0.063 and 0.021, mg/mL, respectively. Due to the presence of phenolic hydroxyls, RUT, NEO, BUD, LIQ, QUE, ISR, KAE and ISL were the primarily antioxidant contributors of *I*.*tinctoria*, which can act as the hydrogen/electron donors to neutralize peroxyl free radicals. In this study, ITHRCs extracts exhibited better antioxidant activities than ITFGRs, which can be partly explained by the higher level of TFL as against ITFGRs. However, one essential difference of traditional Chinese herbal medicines from the synthetic drugs is that their therapeutic effects are due to the joint contribution of multi-components, not only a few ones. Possible synergistic effects of the multiple constituents in ITHRCs extracts should be also taken into considerations. Overall, the antioxidant activity screening results are indicative of the potential of ITHRCs as more effective medicaments compared with naturally occurring roots in food and pharmaceutical industries.

### Predominance of ITHRCs

Although *I*. *tinctoria* can be cultivated as valid economic crops, the quantity of phytochemicals from field grown plants is often fluctuating and heterogeneous due to unfavorable environmental conditions (e.g. seasonal changes, infestation, diseases, and other biotic and abiotic stresses). Herein, switching from culturing intact plants to hairy roots can be considered as an alternative tool for the efficient production of valuable FL, which will hold immense potential for food, agricultural and pharmaceutical applications. This study provided a high-productive ITHRCs (24 day-old) that is capable of biosynthesizing FL in higher yield and quality as against that of 2 year-old field grown roots. Moreover, the proposed system exhibits several potential superiorities as follows: firstly, the uniform quality of products harvested anywhere under the strictly controlled conditions; secondly, the green and sustainable production system irrespective of climate/ecology-related and agrochemical problems; thirdly, the scalable production of products in bioreactor for the commercial purposes.

### Conclusions

The present study is the first report of establishment of ITHRCs for the efficient production of valuable FL. ITHRL V was found to be the lead line and was confirmed by the molecular characterization. Under the optimal conditions (MS/2 medium, temperature 24.7°C, inoculum size 0.75%, sucrose concentration 3.06% and initial pH 5.8), the total FL accumulation in ITHRCs (24 day-old) achieved was 438.10 μg/g DW, which demonstrated the superiority as compared to that of 2 year-old ITFGRs (341.73 μg/g DW). Moreover, LC-MS/MS analysis was performed for the quali-quantitative determination of eight FL constituents (RUT, NEO, BUD, LIQ, QUE, ISR, KAE and ISL) from ITHRCs. Additionally, ITHRCs extracts exhibited superiority in scavenging radicals and inhibiting lipid peroxidation as compared to those of ITFGRs. Overall, the present study highlights the optimization of ITHRCs culture conditions to better predict and define FL biosynthetic capacity, which makes this promising biological system as an attractive platform for the industrial applications or metabolism studies.

## Supporting Information

S1 Table Specific primers employed for PCR and their amplifications programs. (DOC)

S2 Table BBD results for biomass production and FL accumulation during the hairy roots culture process. (DOC)

S3 Table Mass spectrometric parameters for eight FL constituents in ITHRCs. (DOC)

S4 Table ANOVA results of the quadratic models for biomass production and FL accumulation. (DOC)
