# Supplementary material for: Establishment of Hairy Root Cultures by Agrobacterium Rhizogenes Mediated Transformation of Isatis Tinctoria L. for the Efficient Production of Flavonoids and Evaluation of Antioxidant Activities
Source: PLoS One. 2015 Mar 18;10(3):e0119022. doi: 10.1371/journal.pone.0119022 (PMC4364778; doi:10.1371/journal.pone.0119022)
Supplement: S1 Table — (DOC) [file pone.0119022.s001.doc]

**S1 Table.** Speciﬁc primers employed for PCR and their amplifications programs.

| Genes | Primers | PCR program | Products (bp) |
| --- | --- | --- | --- |
| *rol*B | 5-TAGCCGTGACTATAGCAAACCCCTCC-3 (forward) | Initial denaturation at 94 °C for 5 min, followed by 30 cycles of denaturation at 94 °C for 30 s, annealing at 59 °C for 50 s and extension at 72 °C for 50 s, and a final extension at 72 °C for 7 min | 670 |
|  | 5-GGCTTCTTTCTTCAGGTTTACTGCAG-3 (reverse) |  |  |
| *rol*C | 5′-TAACATGGCTGAAGACGACC-3′ (forward) |  | 534 |
|  | 5′-AAACTTGCACTCGCCATGCC-3′ (reverse) |  |  |
| *aux*1 | 5′-TTCGAAGGAAGCTTGTCAGAA-3′ (forward) |  | 350 |
|  | 5′-CTTAAATCCGTGTGACCATAG-3′ (reverse) |  |  |
| *vir*D | 5′-ATGTCGCAAGGCAGTAAGCCC A-3′ (forward) |  | 438 |
|  | 5′-GGAGTCTTTCAGCAGGAGCAA-3′ (reverse) |  |  |
