# Supplementary material for: Establishment of Hairy Root Cultures by Agrobacterium Rhizogenes Mediated Transformation of Isatis Tinctoria L. for the Efficient Production of Flavonoids and Evaluation of Antioxidant Activities
Source: PLoS One. 2015 Mar 18;10(3):e0119022. doi: 10.1371/journal.pone.0119022 (PMC4364778; doi:10.1371/journal.pone.0119022)
Supplement: S2 Table — (DOC) [file pone.0119022.s002.doc]

**S2 Table.** BBD results for biomass production and FL accumulation during the hairy roots culture process ^a^.

| Runs | Factors | | |  | Biomass DW  (g/L) | TFL content  (μg/g) |
| --- | --- | --- | --- | --- | --- | --- |
|  | *X*_1_ (CT ^b^, °C) | *X*_3_ (SC ^c^, %) | *X*_2_ (IS ^d^, %) | *X*_4_ (t ^e^, days) |  |  |
| 1 | -1 (20) | -1 (2) | 0 (0.7) | 0 (24) | 9.07 | 326.1 |
| 2 | 1 (30) | -1 (2) | 0 (0.7) | 0 (24) | 8.59 | 308.5 |
| 3 | -1 (20) | 1 (4) | 0 (0.7) | 0 (24) | 9.35 | 333.3 |
| 4 | 1 (30) | 1 (4) | 0 (0.7) | 0 (24) | 8.91 | 312.1 |
| 5 | 0 (25) | 0 (3) | -1 (0.4) | -1 (18) | 9.43 | 353.2 |
| 6 | 0 (25) | 0 (3) | 1 (1) | -1 (18) | 11.29 | 372.7 |
| 7 | 0 (25) | 0 (3) | -1 (0.4) | 1 (30) | 10.11 | 346.8 |
| 8 | 0 (25) | 0 (3) | 1 (1) | 1 (30) | 10.57 | 363.9 |
| 9 | -1 (20) | 0 (3) | 0 (0.7) | -1 (18) | 9.08 | 328 |
| 10 | 1 (30) | 0 (3) | 0 (0.7) | -1 (18) | 8.72 | 312.5 |
| 11 | -1 (20) | 0 (3) | 0 (0.7) | 1 (30) | 9.17 | 327.1 |
| 12 | 1 (30) | 0 (3) | 0 (0.7) | 1 (30) | 8.73 | 302.4 |
| 13 | 0 (25) | -1 (2) | -1 (0.4) | 0 (24) | 10.15 | 342.9 |
| 14 | 0 (25) | 1 (4) | -1 (0.4) | 0 (24) | 9.52 | 353.1 |
| 15 | 0 (25) | -1 (2) | 1 (1) | 0 (24) | 11.20 | 357.9 |
| 16 | 0 (25) | 1 (4) | 1 (1) | 0 (24) | 10.93 | 360.2 |
| 17 | -1 (20) | 0 (3) | -1 (0.4) | 0 (24) | 8.74 | 303.8 |
| 18 | 1 (30) | 0 (3) | -1 (0.4) | 0 (24) | 7.95 | 290.7 |
| 19 | -1 (20) | 0 (3) | 1 (1) | 0 (24) | 9.33 | 333.9 |
| 20 | 1 (30) | 0 (3) | 1 (1) | 0 (24) | 8.46 | 309.3 |
| 21 | 0 (25) | -1 (2) | 0 (0.7) | -1 (18) | 10.65 | 393.8 |
| 22 | 0 (25) | 1 (4) | 0 (0.7) | -1 (18) | 11.02 | 393.7 |
| 23 | 0 (25) | -1 (2) | 0 (0.7) | 1 (30) | 10.59 | 372.4 |
| 24 | 0 (25) | 1 (4) | 0 (0.7) | 1 (30) | 10.88 | 409.8 |
| 25 | 0 (25) | 0 (3) | 0 (0.7) | 0 (24) | 12.75 | 432.7 |
| 26 | 0 (25) | 0 (3) | 0 (0.7) | 0 (24) | 12.24 | 429.8 |
| 27 | 0 (25) | 0 (3) | 0 (0.7) | 0 (24) | 12.54 | 417.5 |
| 28 | 0 (25) | 0 (3) | 0 (0.7) | 0 (24) | 12.12 | 434.4 |
| 29 | 0 (25) | 0 (3) | 0 (0.7) | 0 (24) | 12.01 | 434.9 |
| 30 | 0 (25) | 0 (3) | 0 (0.7) | 0 (24) | 12.46 | 423.5 |

^a^ ITHRL V in 1/2 MS-based liquid medium (pH 5.8); ^b^ CT is expressed as the culture temperature (°C); ^c^ SC is expressed as the sucrose concentration (%, g/L); ^d^ IS is expressed as the inoculum size (%, g/L); ^e^ t is expressed as the harvest time (days).
