# Supplementary material for: Establishment of Hairy Root Cultures by Agrobacterium Rhizogenes Mediated Transformation of Isatis Tinctoria L. for the Efficient Production of Flavonoids and Evaluation of Antioxidant Activities
Source: PLoS One. 2015 Mar 18;10(3):e0119022. doi: 10.1371/journal.pone.0119022 (PMC4364778; doi:10.1371/journal.pone.0119022)
Supplement: S3 Table — (DOC) [file pone.0119022.s003.doc]

**S3 Table.** Mass spectrometric parameters for eight FL constituents in ITHRCs.

| Parameters | RUT | NEO | BUD | LIQ | QUE | ISR | KAE | ISL |
| --- | --- | --- | --- | --- | --- | --- | --- | --- |
| Nebulizing gas (a.u.) | 12 | 12 | 12 | 12 | 12 | 12 | 12 | 12 |
| Curtain gas (a.u.) | 10 | 10 | 10 | 10 | 10 | 10 | 10 | 10 |
| Collision gas (a.u.) | 6 | 6 | 6 | 6 | 6 | 6 | 6 | 6 |
| Dwell time (s) | 1.5 | 1.5 | 1.5 | 1.5 | 1.5 | 1.5 | 1.5 | 1.5 |
| Ion spray voltage (V) | -4500 | -4500 | -4500 | -4500 | -4500 | -4500 | -4500 | -4500 |
| Ion source temperature (°C) | 300 | 300 | 300 | 300 | 300 | 300 | 300 | 300 |
| Focusing potential (V) | -400 | -400 | -400 | -400 | -400 | -400 | -400 | -400 |
| Entrance potential (V) | -10 | -10 | -10 | -10 | -10 | -10 | -10 | -10 |
| Declustering potential (V) | -66 | -52 | -55 | -55 | -48 | -43 | -31 | -40 |
| Collision energy (V) | -48 | -42 | -18 | -30 | -32 | -34 | -19 | -29 |
| Collision cell exit potential (V) | -16 | -14 | -8 | -10 | -8 | -5 | -4 | -5 |
| SRM (amu) | 609.1 → 300.0 | 609.5 → 301.4 | 591.4 → 283.1 | 255.9 → 119.0 | 301.0 → 151.0 | 315.0 → 300.1 | 285.3 → 183.1 | 255.4 → 118.9 |
