# Supplementary material for: Establishment of Hairy Root Cultures by Agrobacterium Rhizogenes Mediated Transformation of Isatis Tinctoria L. for the Efficient Production of Flavonoids and Evaluation of Antioxidant Activities
Source: PLoS One. 2015 Mar 18;10(3):e0119022. doi: 10.1371/journal.pone.0119022 (PMC4364778; doi:10.1371/journal.pone.0119022)
Supplement: S4 Table — (DOC) [file pone.0119022.s004.doc]

**S4 Table.** ANOVA results of the quadratic models for biomass production and FL accumulation.

| Source | Biomass DW (g/L) | | | TFL content (μg/g) | | |
| --- | --- | --- | --- | --- | --- | --- |
|  | *F* value ^a^ | *P*-value ^b^ | Significance ^c^ | *F* value | *P*-value | Significance |
| Model | 50.95 | < 0.0001 | Significant | 42.55 | < 0.0001 | Significant |
| Lack of Fit ^d^ | 1.02 | 0.5233 | Not significant | 2.56 | 0.1553 *0.4404* | Not significant |
| *R*^2^ | 0.9794 |  |  | 0.9754 |  |  |

^a^ Test for comparing term variance with residual variance; ^b^ Probability of seeing the observed *F*-value if the null hypothecs is true; ^c^ *P*-value less than 0.05 indicate model term is significant; ^d^ Variation of the data around the fitted model.
